# Chemical Sensors Generated on Wafer-Scale Epitaxial Graphene for Application to Front-Line Drug Detection

**DOI:** 10.3390/s19102214

**Published:** 2019-05-14

**Authors:** Mikael Karlsson, Carl Strandqvist, Johnny Jussi, Olof Öberg, Ingemar Petermann, Louise Elmlund, Simon Dunne, Ying Fu, Qin Wang

**Affiliations:** 1 RISE Research Institutes of Sweden AB, Box 1070, SE-164 25 Kista, Sweden; mikael@pamitus.se (M.K.); johnny.jussi@ri.se (J.J.); olof.oberg@ri.se (O.Ö.); qin.wang@ri.se (Q.W.); 2 Pamitus AB, Timotejvägen 1, SE-352 53 Växjö, Sweden; 3 Swedish National Forensic Centre, SE-581 94 Linköping, Sweden; carl.strandqvist@polisen.se (C.S.); louise.elmlund@polisen.se (L.E.); simon.dunne@polisen.se (S.D.); 4 Department of Applied Physics, Royal Institute of Technology, Science for Life Laboratory, SE-171 21 Solna, Sweden; fu@kth.se

**Keywords:** epitaxial graphene, sensors, microfluidics, photoactivity, illicit drugs, forensics

## Abstract

Generation of large areas of graphene possessing high quality and uniformity will be a critical factor if graphene-based devices/sensors are to be commercialized. In this work, epitaxial graphene on a 2" SiC wafer was used to fabricate sensors for the detection of illicit drugs (amphetamine or cocaine). The main target application is on-site forensic detection where there is a high demand for reliable and cost-efficient tools. The sensors were designed and processed with specially configured metal electrodes on the graphene surface by utilizing a series of anchors where the metal contacts are directly connected on the SiC substrate. This has been shown to improve adhesion of the electrodes and decrease the contact resistance. A microfluidic system was constructed to pump solutions over the defined graphene surface that could then act as a sensor area and react with the target drugs. Several prototypic systems were tested where non-covalent interactions were used to localize the sensing components (antibodies) within the measurement cell. The serendipitous discovery of a wavelength-dependent photoactivity for amphetamine and a range of its chemical analogs, however, limited the general application of these prototypic systems. The experimental results reveal that the drug molecules interact with the graphene in a molecule dependent manner based upon a balance of π-stacking interaction of the phenyl ring with graphene (p-doping) and the donation of the amine nitrogens lone pair electrons into the π-π*-system of graphene (n-doping).

## 1. Introduction

Ever since its discovery and isolation by Novoselev and Geim [1], graphene has been studied intensively due to its many interesting properties arising from its two-dimensional sp${}^{2}$-hybridized carbon lattice. The electronic structure from this extended network allows for free movement of electrons through the π and π* bands [2]. Surface interactions of molecules possessing freely moving carriers deposited onto the graphene surface alter the electronic structure of the graphene by acting as electron donors or acceptors to the π-bands [3]. Whilst numerous and broad applications of graphene-based materials have been reported over a wide range of scientific fields [4,5,6,7], the commercial application of graphene will require large-scale fabrication with high quality and uniformity. Epitaxial graphene [8] on silicon carbide (EG-SiC), formed by the sublimation of Si from SiC, is a graphene fabrication method which has previously demonstrated these properties on wafer-scale, making it suitable for industrial applications [9,10]. The use of epitaxial graphene in sensing applications has also been demonstrated where molecular interactions at the surface gave rise to detectable changes in resistivity [7,11].

Within the field of forensics, there is a constant demand for the development of new and more cost-efficient tools for detection of narcotics in the field at both the bulk and trace level. Whilst many different techniques based on presumptive testing, such as chemical ‘spot’ tests [12], colorimetric tests [13] and immunoassays utilizing nanoparticles [14] are available, confirmation using more sophisticated techniques in a laboratory setting (for example gas chromatography with mass spectrometry [15]) are almost always required. Field testing of suspected drug users and seized samples forms a vital link in the chain of evidence/investigation and continues to be an area wherein much work is ongoing, for example, methamphetamine/amphetamine detection in urine by surface-enhanced Raman spectroscopy [16] and illegal drug detection by capillary electrophoresis from oral fluids [17].

In the present work, we describe and demonstrate chemical sensors for illicit drug detection based on epitaxial graphene synthesized on the silicon face of a silicon carbide (SiC) wafer with an application as a forensic field testing apparatus in mind. The devices were characterized in terms of their structural and electrical properties by the transmission line measurements (TLM) method, shear testing and measurements of the current-voltage characteristics. The electrodes of the sensors were processed on the graphene surface with a series of anchors where the metal contacts were in direct contact to the SiC substrate, which increases adhesion of the contacts and decreases the contact resistance due to an increased carrier injection through edge effects [18]. In order for the graphene surface to function as a sensor element, a microfluidic system was developed to allow continuous flow of analyte solutions into the cell and thereby promote interaction with the graphene surface.

Graphene is not expected to display significant differences in sensitivity between closely related analytes and thus in this study antibodies were utilized to establish selectivity. Two different prototypic systems were investigated. In the first, antibodies were immobilized onto magnetic beads to enable their localization upon the graphene surface using an external neodymium magnet positioned directly under the cell. This non-covalent interaction allows magnetic beads immobilized with different antibodies to be easily exchanged within the sensor cell. The second prototype involved the in-situ derivatization of antibodies with 1-pyrene butyric acid-N-hydroxysuccinimide ester (pyrene-NHS) already bound to the graphene surface through non-covalent interactions (π-stacking). The planar hydrophobic pyrene anchor has been used previously to locate biomolecules onto carbon nanotubes and thus can be expected to provide close-contact between the graphene surface and the antibodies in this application. For both of these prototypes exposure to antigen solutions should result in both structural and/or electronic changes in the immobilized antibody, the effects of which are relayed to the graphene, resulting in a detectable change in resistivity. As only a limited number of antibodies with sufficient affinity and selectivity against illicit drug substances were commercially available at the time of this study, amphetamine and cocaine were chosen as representative antigens and a range of monoclonal antibodies against these antigens were purchased. The successes of these prototypes together with the discovery of a light-induced activity of the graphene surface in the presence of the antigens are discussed in detail in Section 3.

## 2. Experimental

### 2.1. Materials and Chemicals

The 1x phosphate buffered saline (PBS) pH 7.4 (97062-732) tablets were purchased from VWR International, Stockholm, Sweden. PBS-Tween (PBS-T, Pierce 20x, 28352), bovine serum albumin (BSA, Blocker 10x in PBS, 37525) and Dynabeads MyOne tosylactivated 1 μm magnetic beads (Cat. No. 65501) were purchased from Fisher Scientific, Gothenburg, Sweden. Ammonium sulphate (A4418), glycine (50046) and 1-pyrene butyric acid-N-hydroxysuccinimide ester (pyrene-NHS, 95%, 457078) from Sigma Aldrich, Stockholm, Sweden were used. All primary antibodies used were monoclonal, produced in mouse. Anti-amphetamine antibodies were purchased from Abcam, Cambridge, United Kingdom (8.F.10.B), Acris antibodies, Herford, Germany (BDI088) and Antibodies-online, Aachen, Germany (M9905225), along with one anti-cocaine antibody from Acris antibodies, Herford, Germany (IP3G2). Amphetamine, methamphetamine, para-methylamphetamine, *n*-ethylamphetamine, phentermine and cocaine were all supplied by the Swedish National Forensic Centre (NFC).

### 2.2. Fabrication of Graphene-Based Device

**Figure 1:**
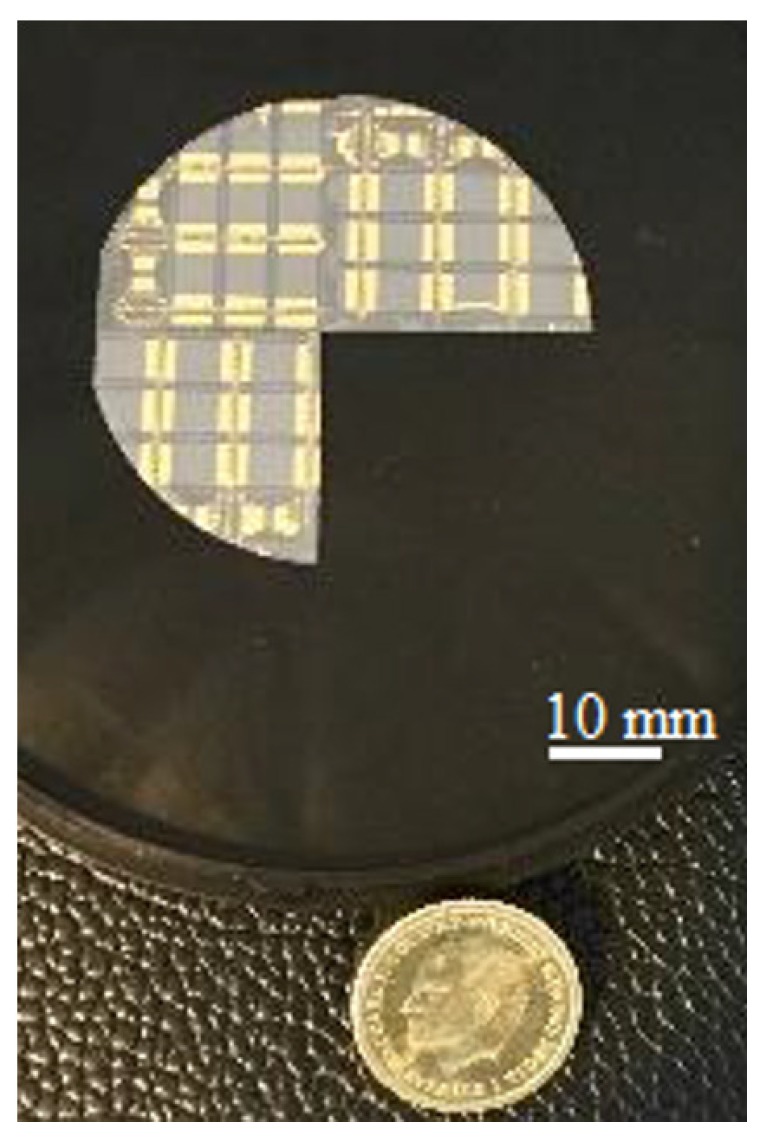
Processed epitaxial graphene on silicon carbide (EG-SiC) wafer with graphene-based devices and different test structures.

**Figure 2:**
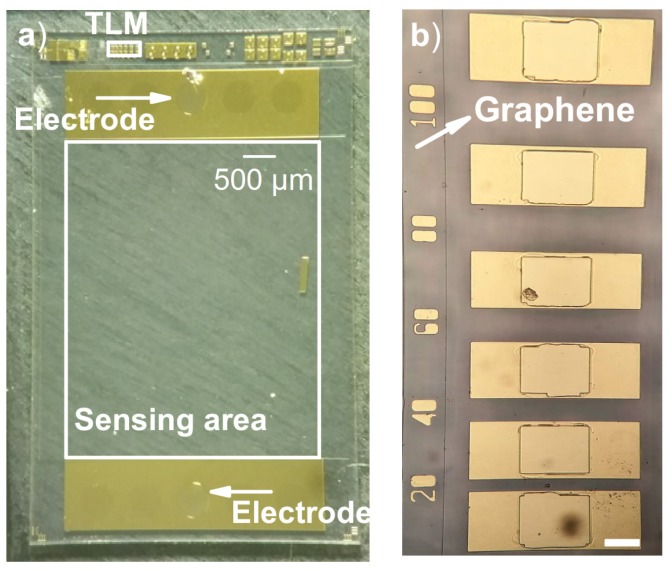
(**a**) Photograph of the graphene-based device, including marked transmission line measurements (TLM) structure. Below electrodes on each side of the device, openings were etched to the SiC substrate before metal deposition, causing the metal contacts to be anchored to the SiC substrate. Except for the middle opening (i.e., electrodes) on each side of the device and the active sensing area, passivation was performed with SU-8; (**b**) closeup of the TLM structure where contacts are separated at different lengths ranging from 20 μm to 100 μm, scale bar of 50 μm.

In short, the device fabrication process consisted of isolating individual active graphene areas by dry etching a 2-inch graphene-on-SiC wafer, forming contacts/electrodes, passivating non-active areas and finally dicing the wafer to chips. The graphene wafer was produced by means of Si sublimation from semi-insulating, Si-face, on-axis, 4H-silicon carbide (SiC) substrates, forming epitaxial graphene on SiC (EG-SiC) [19,20]. The graphene device patterning onto the wafer was performed using standard semiconductor microfabrication technology at RISE’s ISO-9001 certificated clean-room facilities. The graphene active area was defined by photolithography and then reactive-ion etching in O${}_{2}$-plasma. The metal contacts were formed by photolithography, Ti/Au deposition and lift-off, anchored to the SiC substrate by ten round openings per device (five per side with a diameter of approximately 0.25 mm). Passivation of the wafer was achieved using the negative photoresist SU-8, except for a rectangle (length 4.7 mm, breadth 4.8 mm) in the middle of each device and one of the contact openings on each side of every sensing area. The two-inch wafer resulted in 20–24 devices using this process of fabrication, see Figure 1. Figure 2 illustrates a fabricated graphene device with the active sensing area, the electrodes and test structures including a TLM feature as seen in Figure 2b.

### 2.3. Device Characterization

The TLM method [21] was used to evaluate the contact resistance of the fabricated graphene-based devices. The TLM structure consisted of a series of contacts, separated at different lengths ranging from 20 μm to 100 μm, see Figure 2b. The TLM contacts were formed in the same process run detailed in Section 2.2. The TLM structure was characterized by measuring the total resistance $R_{T}$ between each neighbouring pair of contacts with a Keithley 4200A-SCS parameter analyzer. Contact resistance $R_{c}$ is determined from plotting the measured data points as a function of distance between contacts and, assuming ohmic contacts, the y-intercept of a linear fit to the data points yields the contact resistance as $R_{c}=y\left(0\right)/2$. The metal contacts consisted of Ti and Au, which have been used previously for graphene on SiC to provide a contact resistance in the range of 415–655 Ω [22,23,24].

The adhesion quality of the metal contacts was evaluated by attaching bond wires in a loop within the openings using a West-Bond 7476E-79 manual wedge bonder. Destructive and non-destructive shear tests were performed with a DAGE 4000Plus bond tester fitted with a 100 g cartridge. Measurements were carried out by manually lowering a shear tool, inserting the tip into the bond wire loop and applying an upwards force to either a predetermined value of 2.4 g or until the wire broke.

### 2.4. Immobilization of Anti-Amphetamine and Anti-Cocaine Antibodies on Magnetic Beads

To maximize the coverage of the magnetic beads and overall affinity towards the antigen, a mixture of the three anti-amphetamine antibodies was used in a 1:10:10 (8.F.10.B: M9905225:BDI0) ratio. For those beads designed for the detection of cocaine, only a single strain of anti-cocaine antibody IP3G2 was used due to limited commercial availability. Antibody affinities for their respective antigens were tested using standard Dot blot methodology (see Supplementary Information, S.I). The scheme for immobilization followed an adapted form of the protocol given in Invitrogen’s manual [25]. 18.6 μL of the re-suspended tosyl-activated magnetic beads (100 mg/mL) were washed with PBS and then mixed with 55.8 μL of the antibody solution (1 mg/mL). 23.2 μL coating buffer and 3 M ammonium sulphate in PBS were added and the immobilization step incubated with tilted rotation for 48 hours at room temperature (RT). After isolating the beads, 79 μL 0.5% BSA in PBS-T was used to block the remaining tosyl groups on the magnetic beads. The resulting antibody-immobilized beads were washed three times using a storage buffer, 0.1% BSA in PBS-T, followed by dilution to 3.72 mg/mL and storage at 2 °C to 4 °C until use. Scanning electron microscopy (SEM) was further utilized for characterization of the beads. Particle ELISA and absorbance measurements were used to verify the immobilization of the antibodies onto the magnetic beads (for details see Supplementary Information S.II).

### 2.5. Linking of Anti-Cocaine Antibodies to a 1-Pyrene Butyric Acid-N-Hydroxy-Succinimide Ester (Pyrene Anchor)

The anchoring of antibodies onto the graphene surface using pyrene-NHS was performed directly in a polydimethylsiloxane (PDMS) chamber. Pyrene-NHS (375 mg/mL) in EtOH (99.5%) was flowed over the surface for two minutes, followed by EtOH for 10 minutes. Before immobilization of the antibodies, PBS solution (1x pH 7.4) was applied for three minutes. Anti-cocaine antibody solution (1:2000 from 1 mg/mL in PBS) was then passed over the system for 30 minutes and the surface was thereafter washed with PBS for an additional 30 minutes. Atomic force microscopy (AFM) was used to examine the graphene surface under the surface modification process (see Supplementary Information S.I.I).

### 2.6. Amphetamine and Cocaine Detection Measurements

**Figure 3:**
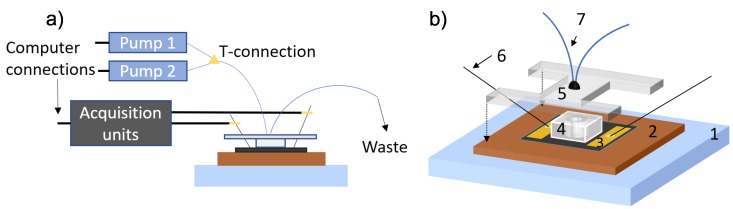
(**a**) Overview of the setup and all connections; (**b**) schematic of the setup with all essential components marked: 1: stage, 2: holder, 3: fabricated graphene-based device, 4: PDMS-based flow chamber, 5: PMMA lid, 6: needle pin probes, 7: microfluidic tubing.

Detection of the drugs was performed with the fabricated graphene-based device using electrical measurements and a flow dynamic system to pump analytes over the active sensing area of the device, as schematically shown in Figure 3a,b. As seen in the figure, the probes are connected to acquisition units, comprised of an Agilent 34970A Data Acquisition/Data Logger Switch Unit, in turn connected to a Keithley 2602A amperometer. The active sensing area is covered with a PDMS-based flow chamber and a poly(methyl methacrylate) (PMMA) lid connected to microfluidic PEEK tubing (IDEX Health and Science LLC.) consisting of inlet and outlet, everything manufactured on site to fit the specific setup. Two Harvard Apparatus 11 pico plus Elite pumps with syringes from AgnTho’s AB are connected to the inlet tubing by a t-connection, which enables the possibility to switch between the two pumps. All measurements were performed at room temperature with a continuous flow of 20 μL/min.

**Figure 4:**
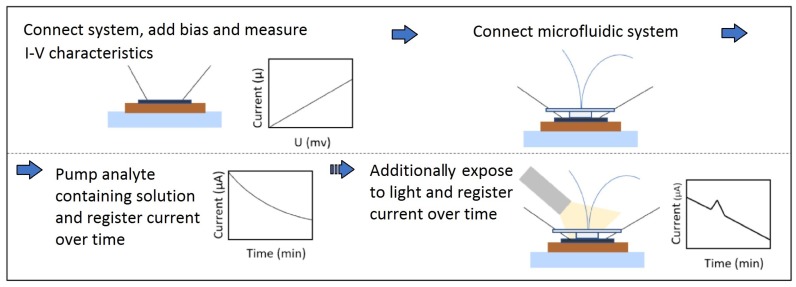
General scheme for the detection of different analytes with the fabricated graphene-based device including additional exposure to light to investigate the discovered photo-physical response.

The general detection method consists of applying a bias to the graphene-based device and probing the openings on either side of the active sensing area. The current over time is further registered when analytes are pumped through the flow system. To detect the analytes pumped through the system, the scheme summarized in Figure 4 was used. After discovering that the investigated analytes showed a photo-physical response, the setup was exposed to the light from a microscope lamp (Schott, KL 2500 LCD) when analytes were pumped through the system. The utilized lamp operates in a color temperature interval between 2600–3000 K, corresponding to a broadband light with a peak position inversely proportional to 1114–966 nm. In some instances, the fluorescent ceiling light was also used in order to cause a photo-physical response. Basically, the scheme in Figure 4 consists of pumping a solution containing an analyte of interest through the system and registering the current over time when the setup is exposed to the microscope lamp.

## 3. Results

### 3.1. Contact Resistance and Mechanical Stability

**Figure 5:**
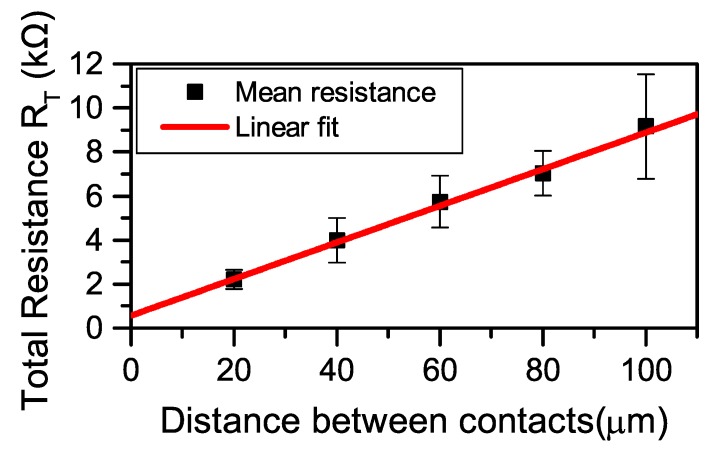
Contact resistance per the TLM method for the fabricated graphene-based devices. The measured resistance as a function of the distance between contacts. Squares represent the mean data points collected from six devices. The red line is the linear fit to the data points and intercepts the y-axis at $R_{T}=2R_{c}$.

The adhesion of metal contacts is of great interest for graphene-based devices in general and is investigated due to it being a limiting factor in terms of the general quality and performance of the devices. Here, eight of the fabricated graphene-based devices were independently measured and characterized with the TLM method for the contact resistance to ensure reproducibility. Each measured graphene-based device yielded a data set with six resistance values, if there was an outlier in the data set, i.e., nonlinear resistance values, or showing non-ohmic characteristics, the whole data set was discarded from the reported results. Out of the measured eight devices, six showed pure ohmic contacts. In Figure 5, the mean values for the measured resistance are shown alongside the linear fit to the results, with a coefficient of determination $R^{2}=99.9\%$ and contact resistance $R_{c}=568/2=284$ Ω.

In total eight metal contacts were tested for mechanical stability, four at 2.4 g in a non-destructive fashion, and four at the maximum required force to break the wire. All contacts passed the set limit for the non-destructive test. The required force to break the respective bonding wires are listed in Table 1. The diversity in resulting forces can generally be accounted by the actual bonding of the wire and the wire itself, rather than the contacts. None of the tests resulted in any physical damage to the contacts. The wire was always the cause of critical failure as verified by visual inspection. If the wire loop was less than perfect, the required breaking force was much lower, as seen for contact #2 (Table 1).

### 3.2. Resistivity Changes

**Figure 6:**
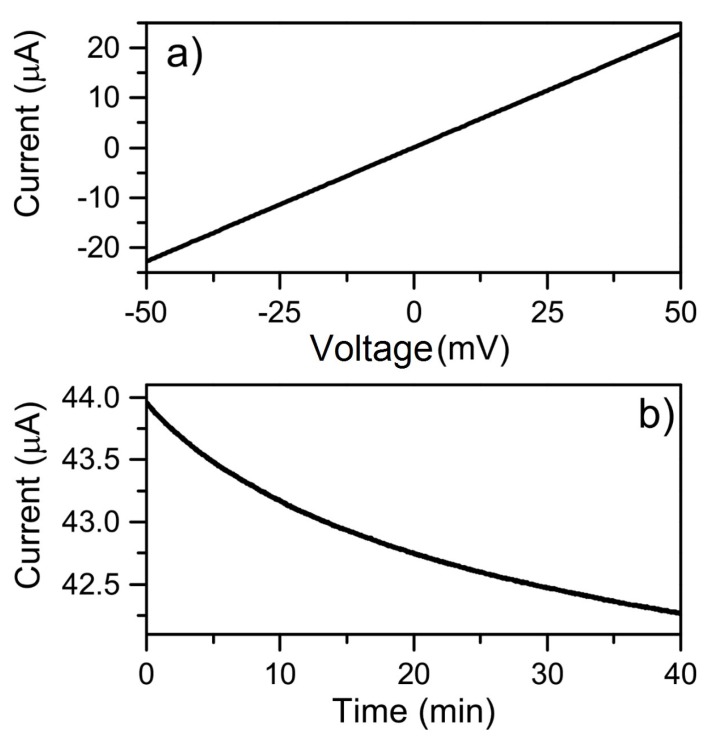
(**a**) Current–potential (I–V) measurement from –50 to 50 mV with no microfluidic system connected; (**b**) time development response with only phosphate buffered saline (PBS) as the pump liquid at 50 mV bias, deionized (DI) water as the pump solution shows the same behaviour.

Current–potential (I–V) measurements between the electrodes on the fabricated-based graphene devices were always performed before any measurement with the flow dynamic system. A typical I–V measurement is shown in Figure 6a wherein a clear linear response and ohmic characteristic can be seen. If a device deviated from such a response, it was not used for further measurements. The effect of the diluent was first investigated in terms of conductivity and photoactivity when in contact with the surface. All experiments with antibodies used PBS as the diluting agent, whilst deionized (DI) water was used for investigations involving solely antigens. Typically, only having PBS or DI water in the flow system produced results as in Figure 6b. The results in the figure is shown for a bias of 50 mV, but measurements were also performed with a bias of 500 mV which gave an overall response of the same nature. The photoactivity was also investigated for both diluents by turning on and off the microscope lamp at different color temperature settings and time points, but there were no measurable changes to the time development response.

#### 3.2.1. Resistivity Changes in the Presence of Surface-Immobilized Antibodies on Magnetic Beads

**Figure 7:**
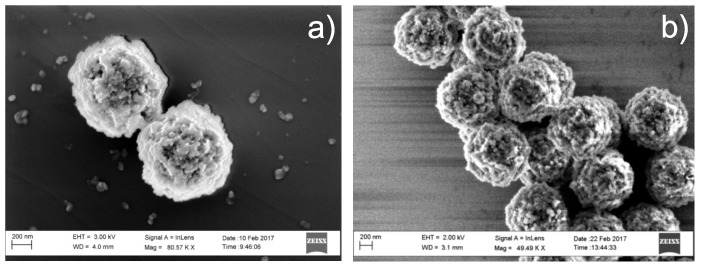
(**a**) Scanning electron microscopy (SEM) image of beads without immobilized antibodies, 86 measurements yielded a mean diameter of 1.03 μm with an interval of 0.88–1.25 μm; (**b**) SEM image of beads with immobilized antibodies, 86 measurements yielded a mean diameter of 1.04 μm with an interval of 0.94–1.14 μm.

In order to characterize the functionalized surface of the magnetic beads, the device surface was thoroughly washed, and the solution phase allowed to evaporate in order to permit SEM experiments. This process was performed on both the native and the functionalized beads in order to examine the structural changes resulting from immobilization of the antibodies. The images revealed a homogeneous morphology and size for the beads, before and after immobilization of antibodies, as well as an increased diameter of approximately 10 nm. Even though the washing and evaporation processes can have disturbed the sensing element-graphene interface it became clear from the SEM images that the magnetic beads were clustered in isolated clumps and not spread as an ideal monolayer (see Figure 7). These results thereby suggest that the reproducible generation of sensor cells using this prototypic system may be difficult.

The first prototypic system utilized antibody-immobilized magnetic beads localized onto the sensor surface using a neodymium magnet. During the measurement of resistivity changes, the flow across the sensor cell was alternated between antigen solutions (both amphetamine and cocaine) and pure PBS solution in order to better elucidate changes in resistivity arising as a result of sensor-antigen events from baseline drift. Despite repeated attempts, no clearly identified responses could be connected to the various flow sequences over the cell when a potential of +50 mV was placed over the graphene.

**Figure 8:**
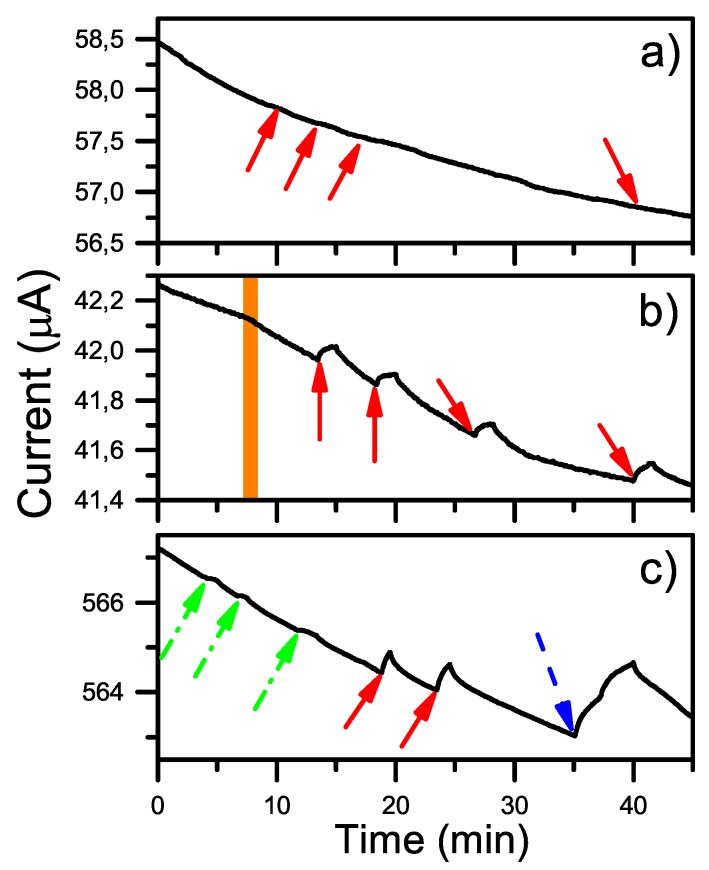
Photoactivity investigation of amphetamine first anchored by and then eluted from antibody coated beads (including exponentially decreasing baselines). (**a**) Only PBS (no analyte) pumped through the system at 50 mV bias; (**b**) amphetamine diluted in PBS pumped through the system at 50 mV bias. The orange marking corresponds to a shift from only PBS to amphetamine in PBS; (**c**) amphetamine diluted in PBS pumped through the system at 500 mV bias. The arrows correspond to the color temperature setting of the microscope lamp: green (dash and dot) = fluorescent ceiling light, red (fully drawn) = 2600 K and blue (dash) = 3000 K.

Figure 8a shows the response of the background PBS flowed with a potential of 50 mV. At one stage during the measurement process a microscope lamp was used to illuminate the sensor cell in order to examine the correct positioning of the needle probes and to examine flow within the microfluidic system. This direct illumination (as opposed to the ambient laboratory fluorescent lighting) resulted in a barely detectable response in Figure 8a. The effect was enhanced when antigen solutions containing amphetamine without magnet beads passed over the graphene surface as shown in Figure 8b. Increasing the potential across the graphene to +500 mV resulted in a significant decrease in resistivity, i.e., increase of the current, as seen in Figure 8c. This response was confirmed using a number of amphetamine analogs in the absence of the magnetic beads.

Due to these findings, a fluidic approach with two steps was established, in which magnetic beads were used for the isolation/selection of antigen on a secondary surface which could later be released to the sensor cell. This demonstrates a method to achieve specificity without competing interactions on the graphene surface (see Supplementary Information S.III).

#### 3.2.2. Photoactivity Induced Resistivity Changes of Amphetamine on Native Graphene Surfaces

As previously discussed in Section 3.2.1, solutions of amphetamine displayed a distinct photoactivity when tested with a modification of the first prototypic system. This effect was heightened when the bias across the graphene sensor was increased to +500 mV and the irradiation source was blue-shifted (3000 K), see Figure 8c. In order to further explore this phenomenon in the absence of the antibodies, the photoactivities of different amphetamine analogs (methamphetamine, *N*-ethyl amphetamine, phentermine, *p*-methyl amphetamine) were studied over time.

**Figure 9:**
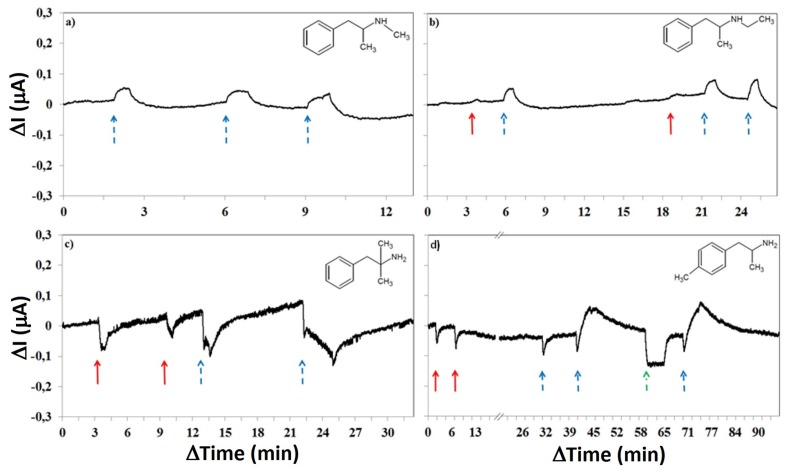
Electrical measurements over time at a bias of 50 mV when different solutions containing amphetamine derivatives were pumped through the system (structural formulas inset). All responses are normalized and baseline-corrected. (**a**) Methamphetamine; (**b**) *N*-ethylamphetamine; (**c**) Phentermine; (**d**) *p*-methylamphetamine. The arrows correspond to the color temperature setting of the microscope lamp; green (dash and dot) = fluorescent ceiling light, red (fully drawn) = 2600 K and blue (dash) = 3000 K.

Figure 9 shows the normalized and baseline-corrected photoactivities of different amphetamine analogs when the bias was set to 50 mV. The baseline response in all cases was, as before, an exponentially decreasing current flow over time. All samples were diluted in deionized water. The colored arrows in the figure both mark when the microscope lamp was turned on, as well as the utilized color temperature setting (red = 2600 K and blue = 3000 K, or green = fluorescent ceiling light).

The *N*-methylated analog, methamphetamine, only showed measureable photoactivity when the microscope lamp was set to 3000 K, no response was seen for 2600 K, see Figure 9a. It was also noted that a longer flow time of methamphetamine solutions through the system was needed before any response could be seen, suggesting that formation of surface interactions are less favorable for methamphetamine than amphetamine.

*N*-Ethylamphetamine displayed similar results to methamphetamine, with a clear increase in the current when exposed to illumination at 3000 K, but with a barely visible increase in current at 2600 K. Compared to methamphetamine, there was however no need for prolonged flow of the amphetamine analog solution through the system before this response became measurable, see Figure 9b.

Phentermine on graphene rendered a decrease in current upon light exposure. With the light set to 3000 K a larger decrease, compared to 2600 K, could be seen as well as a change in slope after the initial response, see Figure 9c.

The current also decreased upon light exposure for *p*-methylamphetamine, but setting the light to 3000 K for an extended time (minutes instead of seconds) gave a rapid increase following the initial decrease, see Figure 9d. This behavior was only observed for p-methylamphetamine. The overall signal from *p*-methylamphetamine can therefore be seen as an increase in conductivity after prolonged light exposure.

#### 3.2.3. Resistivity Changes in the Presence of Surface-Immobilized Anti-Cocaine Antibodies with a Pyrene Anchor

**Figure 10:**
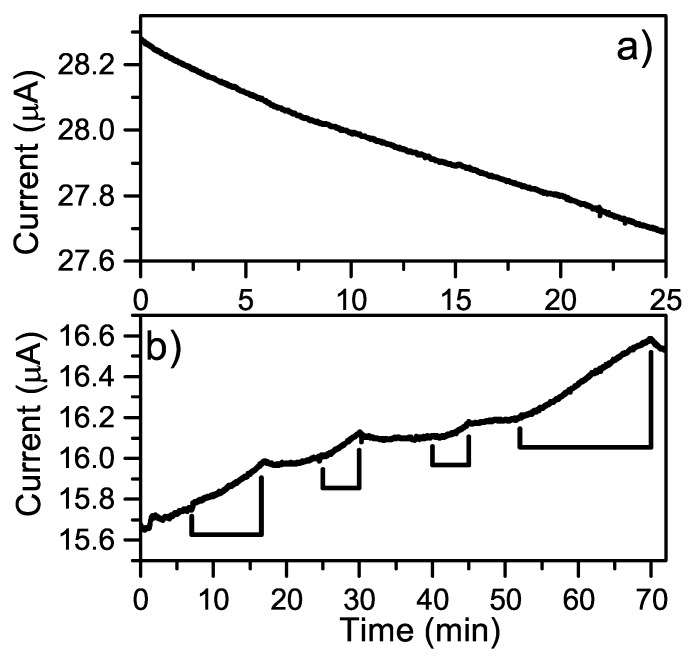
Electrical measurements with cocaine diluted in PBS as the pumping solution with and without the presence of pyrene-N-hydroxysuccinimide ester (NHS)-linked antibodies (**a**) without pyrene-NHS-linked antibodies; (**b**) with pyrene-NHS-linked antibodies, where the indicators show when cocaine in PBS is used as the pumping solution.

Electrical measurements were performed for a solution containing cocaine diluted in PBS and investigated for the conductivity and photoactivity following the scheme in Figure 4. However, without any form of modification to the original detection scheme, there were no comparable differences in terms of the conductivity to when the pumping solution only contained PBS, as seen in Figure 10a and Figure 6b. Furthermore, no photoactivity response was observed in any of the measurements despite testing several color temperature settings of the microscope lamp. This negligible photoactivity demonstrated by cocaine solutions on the graphene surface led to the choice of cocaine as the antigen for the second prototypic system. The steric bulk due to the bicyclic structure of cocaine may be the underlying reason for its lower activity.

Pyrene-NHS was allowed to interact with the graphene surface followed by the linkage of anti-cocaine antibodies as described in Section 2.5. From the results presented in Figure 10b it is evident that upon changing the pumping solution from PBS to cocaine in PBS, there is a gradual increase in the conductivity during the whole time frame. All instances of changing the pump solution back to PBS caused an initial decrease in conductivity, followed by a plateauing of the conductivity to an equilibrium state.

## 4. Discussions

Our findings in this study show that the EG-SiC was formed with low contact resistance and processed with metal contacts of high adhesion capabilities. The specific processing of the metal contacts anchored to the substrate layer could prove to be vital for future prospects, especially for applications where a low contact resistance is required as for instance in high-frequency applications [24,26].

Two prototypic systems incorporating the non-covalent anchoring of antibodies (specific against amphetamine and cocaine respectively) to the graphene surface were tested. Both systems possessed design elements that could be utilized in future prototypes. Whilst difficulties were encountered in achieving a monolayer of magnetic beads (and thus a reproducible device), the use of immobilized antibodies to capture and release antigens in a preselection step has been demonstrated. The attachment of antibodies using a planar hydrophobic pyrene anchor in the second prototype provided promising results for cocaine, demonstrating that binding events between antigen–antibody can be relayed to the graphene and monitored electrically. The mechanism resulting in resistivity changes for this prototype is currently under study to examine if the observed effects are due to changes in strain, conformation, electrical changes, solvation etc. However, due to the later-detected photoactivity of amphetamine analogs, the generality of this second prototype is limited since the small molecular size of these antigens allows their ready access to exposed graphene surfaces and thereby a competing process of resistivity-changing events.

Since the detection scheme involved alternated pumping of the different solutions with and without analyte and with and without light exposure in an otherwise unchanged environment, possible spurious effects on the measured current by e.g., temperature fluctuations could be excluded.

All amphetamine analogs demonstrated a wavelength-dependent photoactivity with a higher activity when irradiated with a microscope lamp set at 3000 K compared to 2600 K. Adsorption of organic molecules onto graphene has been studied both theoretically and experimentally, resulting in the conclusion that the photoinduced charge carriers such as electrons or holes depending upon the adsorbates can interact actively with graphene [27,28,29,30,31,32]. The amphetamine analogs in this study were chosen to represent a variety of alkyl substitution patterns on and around the amine nitrogen. All analogs except *p*-methyl-amphetamine possessed an unsubstituted phenyl group β to the amine nitrogen. It has been shown that amines and aryl compounds can interact with graphene’s electronic structure when adsorbed non-covalently (physisorption) and these groups were expected to dominate surface interactions for these analogs. Increased alkylation around these donor/acceptor groups should therefore sterically hinder the formation of a charge transfer complex at the surface [27,29,32].

Both methamphetamine and *N*-ethylamphetamine gave similar responses to amphetamine, although considerably weaker than for the parent compound. The overall response for these three analogs was an increase in conductivity when a solution of the analog was flowed over the graphene surface most likely as a result of the amine nitrogen acting as an electron donor.

Irradiation of the sensor when a solution of phentermine was flowed across the graphene surface resulted in a decrease in conductivity for the graphene surface. Phentermine possesses a crowded primary amine functionality with α,α-dimethyl substitution at the hydrophilic end of the molecule, which may inhibit the nitrogen from acting as an electron donor. This would suggest that formation of a light-induced π-complex between phentermines phenyl ring and graphene results in a disruption of conductivity within the graphene surface (increase in resistivity).

The *p*-methylamphetamine displays an initial reduction in conductivity (π-complex formation) upon light exposure followed by an increase in conductivity (donation of electron density from the nitrogen atom). The slower formation of a weaker π-complex (resulting from the methyl substitution on the phenyl ring) may lead to the separation of these two events in time. Studies have shown that the correct orientation of the amine functionality is vital for a strong interaction with graphene’s electronic structure [30,32]. Future testing of this phenomenon with additional analogs is required to fully understand it.

## 5. Conclusions

In this work, batch fabricated graphene devices with low contact resistance were utilized for detecting illicit drugs by investigating the interaction between graphene and drug molecules, which are of special interest not only in the criminal justice system but also for the health care industry.

The use of magnetization as a means of localizing the sensor element upon the graphene surface proved difficult due to bead clustering. The combination of magnetic beads and antibodies could however be utilized for the preselection of analytes (antigens) before interaction with the graphene surface, thereby creating high substance-specificity even for complex mixtures of active substances and cutting agents.

The use of immobilized antibodies on the graphene surface via a hydrophobic pyrene anchor proved promising as long as the antigen itself showed no photoactivity with the surface. Antigen–antibody binding events relayed from the antibody through the butyric amide linker to the pyrene anchor and ultimately to the graphene surface resulted in resistivity changes.

The serendipitous discovery of photoactivity between amphetamine analogs and graphene rendered the viability of the two initial prototypic systems conjectural. The intensity of these photo-responses was found to be wavelength-dependent. The nature of the overall response is conjectured to arise from a balance between π-stacking and interactions of the amine group with the surface. However at this stage the possibility of light-facilitated chemical oxidation events cannot be eliminated.

The combination of preselection of analyte using immobilized antibodies with detection using a native EG surface shows promise as the technology behind hand-held devices utilizing batch-fabricated graphene-based sensors for rapid front-line detection of illicit drugs.

## Figures and Tables

**Table 1:** Adhesion measurements showing the required force to break the bonding wire.

Contact #	Force (g)
1	6.70
2	4.70
3	7.94
4	9.36

## Supplementary Materials

The following are available online at https://www.mdpi.com/1424-8220/19/10/2214/s1.
