# Supplementary material for: Chemical Sensors Generated on Wafer-Scale Epitaxial Graphene for Application to Front-Line Drug Detection
Source: Sensors (Basel). 2019 May 14;19(10):2214. doi: 10.3390/s19102214 (PMC6567372; doi:10.3390/s19102214)
Supplement: Supplementary file 1 [file sensors-19-02214-s001.pdf]

Supplementary Information

S.I Dot blot

S.I.I Materials and procedure

Dot blot was performed with the three anti-amphetamine antibodies (8.F.10.B, M9905225, BDI0) and the one anti-cocaine antibody (IP3G2), investigating their affinity for their respective antigens and how different ratios would affect the ligand-binding process. Cocaine and amphetamine were dissolved in PBS, 10.5 mg amphetamine and 10.7 mg cocaine to 100 µL PBS respectively giving at least 100 mg/mL. To increase solubility the antigen solutions were heated to 37 °C for 30 minutes. Dot-blot membrane of nitrocellulose (0.2 µm, Bio-Rad #1620252) was cut into small squares and placed in the lids of Eppendorf tubes. On the pieces of membrane 2 µL of the respective antigen solutions was applied and left to dry for 10 minutes. The membranes were then incubated in 100 µL blocking buffer, 0.1 % BSA in TBS-T (Tris Buffered Saline – Tween, T9039 Sigma), on a shaker for 30 minutes.
The primary antibodies were diluted from their respective stem solution, 1 mg/mL in PBS, in ratios of 1:100, 1:1000, 1:10000 and 1:50000 rendering concentrations of 10, 1, 0.1 and 0.02 µg/mL. The blocked membranes were incubated in 100 µL of the different rationed antibody solutions for 30 minutes, after which they were washed with 3x100 µL TBS-T during 3x5 minutes. Negative references did not have any primary antibody added, they were first incubated with the buffer used and then blocked with BSA. The membranes were lastly incubated with 100 µL secondary antibody (Abcam ab97046) solution, 1:1 000 and 1:5 000 from 1 mg/mL respectively.
After washing, the reaction with the horseradish peroxidase (HRP) tagged secondary antibody was performed using ECL-reagent (Optiblot ECL Detect Kit, Abcam ab133406) mixed 1:1 from hydrogen peroxide and luminol solutions for 1 minute before the assay was developed using a CCD camera.

S.I.II Results

Both the blotting of the primary antibodies and the negative references rendered ambiguous results, but still showed the affinity of the primary antibodies. As can be seen in Figure I, it was not always the highest concentration of primary antibody that gave the largest response. The concentration of secondary antibody that rendered the best results was the dilution of 1:1000.
In summary the dot blot experiments founded the selection of a mixture of the three anti-amphetamine antibodies in a ratio of 1:10:10 (8.F.10.B: M9905225 : BDI0) to be used for all of the investigations to follow. The mixture

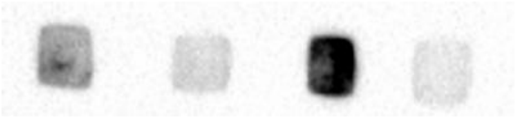

Figure I Dot blot results from 8.F10.B. Concentrations from left to right: 10, 1, 0.1 and 0.02 µg/mL. Secondary antibody 1:1000.

aimed at an increased binding when using the affinity of three clones, their ability to immobilize on the magnetic beads and the presumed variance in sturdiness.

S.II Particle-ELISA and absorbance

S.II.I Materials and procedure

Particle-ELISA and absorbance measurements were used to evaluate the immobilization of antibodies on the magnetic beads, where particle-ELISA investigated the magnetic beads and absorbance of the reaction solution the difference in antibody concentration before and after the immobilization.

Particle-ELISA was performed using the same scheme as in dot blot, but instead of membranes the primary antibodies (S.I.II, 1:10:10 mixture) were immobilized on the beads (never adding any antigen) and the preparative steps were taken in solutions. 500 µL (5 µg/µL), respectively, of beads with and without immobilized antibodies were divided in Eppendorf tubes and put on a magnet for 2 minutes. The supernatant was removed and 499 µL TBS-T (S.II.I) added to each sample. To 100 µL blank (the immobilization step without beads, with primary antibody) and null reference (buffers and reagents from the immobilization step) 399 µL TBS-T was added respectively. All samples, blank and reference were incubated with 1 µL secondary antibody (S.II.I), 1:5000, for 30 minutes on a tilting table.

Following binding with secondary antibody the tubes were put on a magnet, the supernatant removed and the samples, blank and reference washed with 3x500 µL TBS-T and 1x500 µL TBS (Sigma T5912-1L) for 5 minutes on a tilting table respectively. After washes 300 µL TMB (below) substrate was added and incubated for about 3 minutes, thereafter an equal volume of 1 M H_2SO_4 stopped the reaction. The absorbance at 450 nm for all samples, blank and reference was read in a 96-well plate.

The TMB substrate was prepared from 12 mL 0.11 M sodium acetate (Thermo Fisher), 200 µL TMB stock pH 5.5 (3,3',5,5'-Tetrametylbensidin, Sigma) and 10 µL H_2O_2 (Abcam).

S.II.II Results

450 nm	1	2	3	4	5	6	7	8	9
A	3,525	3,513	3,572	3,489	3,479	3,515	3,321	3,441	3,367
B	0,059	0,077	0,096	0,044	0,038	0,060	0,072	0,076	0,092
C	-0,001	0	0	0,004	0,012	-0,001	-0,002	-0,002	0,005
D	0,086	0,095	0,097	0,102	0,086	0,084	0,06	0,06	0,063
E	0,051	0,051	0,057	0,044	0,043	0,048	0,042	0,115	0,108
F	0	0,001	0						

Table SI Results from the particle-ELISA after subtraction of background.

After subtraction the background, where only H_2SO_4 and TMB substrate were mixed (row F), the absorbances are presented in Table SI. For each sample triplicates were prepared and for each tube in the triplicate applications were made in three wells (Figure II), A1-3 is three applications from the same tube and A1-3, 4-6 and 7-9 constitutes one triplicate in the preparation.
Row A has samples with primary antibodies immobilized on magnetic beads, B beads without antibodies, C null sample from buffers only, D beads with primary antibody without addition of the secondary antibody and E beads without primary and secondary antibodies.

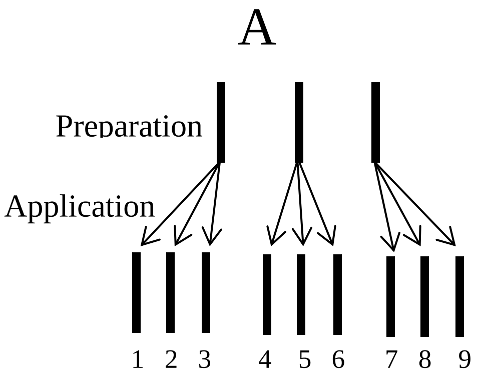

Figure II Sample A prepared in a triplicate and each sample in the triplicate is applied in three wells.

The absorbances at 280 nm (tryptophan) of the primary antibody solution before immobilization on magnetic beads and of the supernatant after the immobilization on beads were performed to measure the difference in concentrations and thereby an estimate of the quantity immobilized on the beads. The measurements were carried out in a NanoDrop, requiring ca 2 µL samples.

280 nm	Abs
A	0
B	1,14
C	1,13
D1	0,65
D2	0,67
D3	0,69
E1	0,12
E2	0,04
E3	0,01
F1	0,02
F2	0,03
F3	0

280 nm	Abs
A	-0,02
B	1,12
C	1,11
D1	0,63
D2	0,65
D3	0,67
E1	0,10
E2	0,02
E3	-0,01
F1	0,00
F2	0,01
F3	-0,02

Table SII Results from the absorbance measurements at 280 nm. Left: raw data, right: after background (F1-3) subtraction.

The measured absorbances, Table SII, were taken for the immobilization step without primary antibody (A), stem solutions of primary antibody 1 mg/mL (B-C), reaction solutions with 0.707 mg/mL primary antibody (D1-3), supernatant after immobilization of primary antibody (E1-3) and the supernatant from the immobilization step without primary antibody (F1-3).
The results from particle-ELISA and absorbance measurements were utilized in order to enhance the immobilization protocol and also for the development of plans for further investigations presented earlier in this work.

S.III Photoactivity of amphetamine with pre-selection
In order to ensure that the observed photoactivity was due to the analyte present in the solution and not due to an artifact of the system, measurements were performed where a pre-selection of the analyte was accomplished using antibody-immobilized magnetic beads on a polytetrafluoroethylene (PTFE) surface.

While amphetamine displayed a photo-induced activity on graphene, the surface in itself cannot be expected to display appreciable selectivity between closely related substances. The use of antibodies immobilized on magnetic beads introduces selectivity before interaction with the graphene surface. In order to investigate this possibility, the microfluidic system was expanded to include a selection/extraction step using anti-amphetamine coated magnetic beads held down by a magnet on the PTFE surface. The antibody-bead coated PTFE surface was exposed to amphetamine in PBS and then washed with PBS, after which it was connected to the graphene-sensor microfluidic system. To elute the amphetamine, 100 mM glycine in PBS with an adjusted pH to 2.5

(HCl) was used. The eluted amphetamine could then be detected on the graphene surface via its photo-induced activity.
